# The correlation between maternal age, parity, cardiac diastolic function and occurrence rate of pre-eclampsia

**DOI:** 10.1038/s41598-021-87953-x

**Published:** 2021-04-23

**Authors:** Dan Zhu, Weiyu Chen, Yuchen Pan, Tingcui Li, Ming Cui, Baoxia Chen

**Affiliations:** Department of Cardiology, NHFPC Key Laboratory of Cardiovascular Molecular Biology and Regulatory Peptides, Peking University Third Hospital, 49 Huayuan North Road, Haidian District, Beijing, 100191 China

**Keywords:** Cardiology, Cardiovascular diseases

## Abstract

To evaluate the effect of age and parity on maternal cardiac diastolic function in second trimester among pregnant women with normal left ventricular ejection fraction. To analyze the correlation between impaired diastolic function and pre-eclampsia. It had been suggested that maternal cardiac adaptations during pregnancy differed between nulliparous and primiparous women and also varied according to age. Impaired cardiac function may precede pre-eclampsia. Therefore, we examined the effects of parity and age on cardiac diastolic function during pregnancy and whether impaired diastolic function during the second trimester correlates with pre-eclampsia. Women with singleton pregnancies at 13 + 0 to 27 + 6 weeks’ gestation and left ventricular ejection fraction (LVEF) ≥ 50% were retrospectively identified. Diastolic function parameters were assessed using transthoracic echocardiography. Pre-eclampsia was identified from medical records. The effect of age and parity on maternal cardiac diastolic function as well as the correlation between impaired diastolic function and occurrence rate of pre-eclampsia were examined. 376 pregnant women were included (median age: 30 years; median gestational age: 14 weeks; 171 primiparous women). LVEF was 66%. Impaired cardiac diastolic function was seen in 7.8% of pregnant women < 35 years compared with 28.6% of those ≥ 35 years (p = 0.000). ROC curve showed women with maternal age over 32 began to have a higher rate of impaired cardiac diastolic function (AUC = 0.704, p = 0.000, sensitivity = 54.5%, specificity = 75.3%). There was no difference in diastolic function indices between maternal women grouped by parity. Higher maternal age was an independent risk factor of declining Em (p < 0.05). Em < 13 cm/s was significantly associated with pre-eclampsia occurrence (HR 8.56; 95% CI 3.40–21.57) after being adjusted for confounders. Maternal age is an independent risk factor for diastolic function decline. There is no difference in cardiac diastolic function between nulliparous women and primiparous women. Pre-eclampsia occurrence is significantly higher in patients with impaired diastolic function at mid-gestation. The application of risk grading using diastolic function at mid-gestation may improve the survival outcomes of pregnant women.

## Introduction

Maternal cardiovascular disease is the major cause of maternal death during pregnancy in western countries^1^. In China, Pregnancies with cardiovascular disease also rank the first among the death of maternal women^2^. Hypertensive disorders are the most frequent cardiovascular disorders during pregnancy and remain a major cause of maternal, foetal, and neonatal morbidity and mortality^1^.

During normal pregnancy, physiological cardiovascular adaptations occur in order to meet demands of the rapidly developing fetus. These cardiovascular adaptations include an initial fall in systemic vascular tone with the purpose of increasing the cardiac output and an expanding plasma volume^1^. Maternal cardiovascular maladaptation is strongly correlated to pregnancy outcome. For example, it has been demonstrated that maternal systolic function adapts worse even at the first trimester in pregnancies that suffer from hypertension complications, early delivery and lower birth weights^3^. It is thus evident that the study of maternal cardiovascular adaptation during pregnancy provides an insight into the interaction between maternal and fetal homeostasis. Impairment of diastolic function of the left ventricle (LV) precedes systolic dysfunction in the development of most cardiac diseases^4^, and it might be an indicator which can predict the risk of pregnancy complication during pregnant period. For instance, studies showed that diastolic dysfunction might be associated with the onset and the severity of pre-eclampsia^5,6^. It is also suggested that adaptations suboptimal to pregnancy were associated with maternal age, parity and other demographic factors such as height, weight, body surface area and ethnicity^7^. Study showed maternal age was a significant predictor of cardiovascular function such as cardiac output, total peripheral resistance, stroke volume, ventricular ejection time^8^. In this study, we investigated the effect of age and parity on maternal diastolic function in second trimester among pregnant women with normal left ventricular ejection fraction. We also analyzed the correlation between impaired diastolic function in the second trimester and the occurrence rate of pre-eclampsia in the whole period of gestation.

## Method

In this retrospective study, we identified consecutive singleton pregnant women with normal left ventricular ejection fraction within the range of 13 + 0 and 20 + 6 weeks of gestation who were diagnosed by echocardiography at Peking University third hospital from January 2017 to July 2017. Subjects with any of the following criteria were excluded: endocrine diseases such as diabetes mellitus, thyroid disorder; cardiovascular disease such as coronary heart disease, hypertensive disorders, cardiomyopathy, valvular disease and arrhythmia; autoimmune disease; respiratory disease; hematological disease; liver disease; kidney disease; neurological disease; and tumor; International Classification of Diseases(ICD)-9 and -10 codes were used for identifying patients’ demographics. Women were tracked until the diagnosis of pre-eclampsia was made or child birth, at which time they were surveyed. Patients were classified into four groups according to age and parity. Advanced maternal age was defined as over 35 years old. Nulliparous women were defined as those with no previous live births. Primiparous women were those with one live birth before the index delivery. History of abortion included both history of induced abortion and miscarriage. Pre-eclampsia was defined as gestational hypertension accompanied by one or more of the following new-onset conditions at or after 20 weeks of gestation: (1) proteinuria, (2) Other maternal organ dysfunction, including: acute kidney injury; liver involvement; neurological complications; hematological complications (3) Uteroplacental dysfunction^9^. Pre-eclampsia was considered severe if one or more of the following criteria is present: (1) systolic blood pressure (SBP) over 160 mmHg and/or diastolic blood pressure (DBP) over 110 mmHg; (2) proteinuria over 5 g in 24-h urine or over 3+ in two random urine samples collected at least 4 h apart; (3) oliguria less than 500 ml in 24 h; (4) cerebral or visual disturbances; (5) pulmonary edema or cyanosis; (6) epigastric or right upper-quadrant pain; (7) impaired liver function; (8) thrombocytopenia(platelet counts, 100,000/mm^3^); (9) fetal growth restriction. Office blood pressure was measured by the same type of electronic sphygmomanometer in rest condition when patients arrived at the outpatient department. Laboratory tests were performed by clinical laboratory department of Peking University third hospital. The Peking University third hospital Institutional Review Board approved the study, and informed consent was obtained from all subjects. All methods were performed in accordance with relevant guidelines and regulations.

### Echocardiography

Echo-Doppler data were obtained using commercially available ultrasonography systems. Left ventricular ejection fraction (LVEF) was calculated by the modified Simpson or the modified Quinones method. LV mass index was calculated by the Devereux formula and indexed for body surface area. The degree of aortic and mitral regurgitation was classified according to the guidelines of the American Society of Echocardiography^10^. Calculation of relative wall thickness was done by the formula: (2 × posterior wall thickness)/(LV end-diastolic diameter). Diastolic function was assessed by mitral inflow velocity (early [E] diastolic velocity and atrial [A] velocity), peak early diastolic myocardial velocity(Em) of the medial and the lateral mitral annulus, E/Em, Left Atrium Volume Index (LAVI) and tricuspid regurgitation velocity according to the guidelines. LAVI was calculated using the area-length method by tracing in both the apical 2- and 4-chamber view.

### Statistical analysis

Continuous variables were summarized as a mean ± SD (standard deviation) or median (25th, 75th percentile). Categorical variables were presented as counts (%). A two-tailed *p*-value < 0.05 was considered statistically significant. For two-group comparison, parametric (2-sample *t*-test) and non-parametric tests (Wilcoxon rank sum test) for continuous variables, and the χ^2^ test or Fisher exact test for nominal variables were adopted.

Multivariable Cox proportional hazard model was applied to assess whether demographic parameter could independently predict diastolic function decline and whether Em could independently predict pre-eclampsia. Clinical and echocardiographic variables were selected based on clinical importance. The Hazard Ratios and 95% confidence intervals (CI) were reported. Penalized smoothing spline (P-splines) was applied to illustrate the correlation between Em and the hazard ratio using univariate cox proportional hazards method for assessing the tendency of the occurrence rate of pre-eclampsia. 10% of Em value (13 cm/s) was used as references. Data were analyzed using SPSS (version 20.0, IBM Corp.).

## Results

376 pregnant women (205 nulliparous women and 171 primiparous women) were finally included into the study analysis. Women’s clinical characteristics were shown in Table 1. Median age was 30 years, 45% were primiparous. Echocardiographic parameters were shown in Table 2. Mean LAVI was 30 ml/m^2^, mean LVEF was 66% and mean value of Em was 17.5 cm/s. We defined deciles of Em (13 cm/s) as impaired diastolic function. There were 44 (11.7%) patients with Em < 13 cm/s. The mean age of primiparous were significantly higher than nulliparous women and the primiparous women had higher Body Mass Index (BMI) and body surface area (BSA) (Table 1). Blood pressure at baseline and during follow-up were showed in Table 1, no difference was found between group divided by age, parity or onset of pre-eclampsia at baseline. as well as Pregnant women over the age of 35 had larger left atrial diameter and area, higher left atrial volume index, larger right atrial area, higher A velocity, lower Em velocity and higher E/Em ratio (Table 2). Primiparous women had larger left atrial dimension (LAD) and left atrial area (LAA), larger right atrial area (RAA) and higher right ventricular end diastolic area (RVEDA) (Table 2).

**Table 1:** Baseline patient characteristics.

Parameter	Maternal age < 35 (n = 306)	Maternal age ≥ 35 (n = 70)	*p*	Nulliparouswomen (n = 205)	Primiparouswomen (n = 171)	*p*	No pre-eclampsia (n = 351)	Pre-eclampsia (n = 25)	*p*
Maternal age, years	28.4 ± 3.2	37.2 ± 1.5	< 0.001	28.0 ± 3.8	32.6 ± 3.9	< 0.001	30.0 ± 4.5	30.6 ± 4.4	0.497
Gestational age, weeks	14 (14, 15)	14 (14, 15)	0.642	14 (14, 15)	14 (14, 15)	0.931	14 (14, 15)	14 (14, 14)	0.164
Height, cm	161.4 ± 5.0	161.4 ± 4.6	0.968	161.3 ± 4.9	161.4 ± 4.9	0.897	161.3 ± 4.9	161.7 ± 5.0	0.968
Weight, kg	60.2 ± 10.2	63.7 ± 9.0	0.180	58.4 ± 10.5	63.3 ± 9.0	< 0.001	60.4 ± 10.0	65.8 ± 8.9	0.012
BMI, kg/m^2^	23.1 ± 3.7	24.4 ± 3.3	0.120	22.4 ± 3.7	24.3 ± 3.4	< 0.001	23.1 ± 3.6	25.2 ± 3.3	0.010
BSA, m^2^	1.60 ± 0.14	1.65 ± 0.13	0.030	1.58 ± 0.15	1.64 ± 0.13	< 0.001	1.6 ± 0.1	1.7 ± 0.1	0.018
Heart Rate, bpm	82.1 ± 10.4	81.7 ± 12.0	0.759	81.9 ± 10.8	82.2 ± 10.6	0.765	81.5 ± 10.6	88.9 ± 9.8	0.001
Baseline SBP, mmHg	113 (108, 121)	117 (110, 123)	0.081	112 (107, 121)	115 (109, 121)	0.063	115 (108, 121)	116 (107, 123)	0.537
Baseline DBP, mmHg	75 (71, 79)	75 (71, 79)	0.964	75 (71, 78)	76 (70, 79)	0.912	76 (71, 79)	74 (71, 78)	0.338
Follow-up SBP, mmHg	114 (108, 122)	117 (112, 123)	0.165	114 (107, 122)	118 (110, 123)	0.056	114 (108, 121)	157 (152, 171)	< 0.001
Follow-up DBP, mmHg	76 (71, 80)	76 (71, 81)	0.744	76 (71, 80)	77 (71, 81)	0.847	76 (71, 79)	109 (104, 116)	< 0.001
History of abortion, count (%)	114 (37.3%)	36 (51.4%)	0.029	63 (30.7%)	87 (50.9%)	< 0.001	139 (39.6%)	11 (44.0%)	0.664

Gestational age represented the time Echocardiography was measured. Follow-up SBP and DBP were defined as the highest blood pressure collected during the follow-up.

Values are given as mean ± standard deviation, median (interquartile range) or count (percentage).

*BMI* body mass index, *BSA* body surface area, *SBP* systolic blood pressure, *DBP* diastolic blood pressure.

**Table 2:** Baseline echocardiographic parameters.

Parameter	Maternal age < 35 (n = 306)	Maternal age ≥ 35 (n = 70)	*p*	Nulliparouswomen (n = 205)	Primiparouswomen (n = 171)	*p*	No pre-eclampsia (n = 351)	Pre-eclampsia (n = 25)	*p*
LAD, cm	30.5 ± 3.6	31.9 ± 2.9	0.005	30.0 ± 3.2	31.4 ± 3.7	< 0.001	30.6 ± 3.5	31.7 ± 3.7	0.155
LAA, cm^2^	14.9 ± 1.7	15.9 ± 1.8	< 0.001	14.7 ± 1.7	15.5 ± 1.8	< 0.001	15.1 ± 2.3	15.4 ± 1.7	0.392
LAVI, ml/m^2^	29 ± 6	32 ± 7	0.006	29 ± 6	30 ± 6	0.076	30 ± 9	29 ± 5	0.614
RAA, cm^2^	12.9 ± 1.6	13.6 ± 1.7	0.006	12.8 ± 1.6	13.3 ± 1.6	< 0.001	13.0 ± 1.7	13.8 ± 1.8	0.315
LVEDA, cm^3^	44.0 ± 5.2	44.9 ± 4.9	0.176	43.8 ± 4.7	44.6 ± 5.7	0.179	44.1 ± 5.3	44.8 ± 2.7	0.506
RV-ap, cm	19.8 ± 3.7	20.1 ± 2.0	0.435	19.4 ± 2.7	20.3 ± 4.1	0.034	19.9 ± 3.5	19.4 ± 2.1	0.563
RVEDA, cm^3^	28.1 ± 4.1	28.3 ± 2.7	0.769	27.8 ± 4.0	28.6 ± 3.6	0.034	28.2 ± 3.9	27.8 ± 1.9	0.650
LVEF, %	65.4 ± 6.5	66.1 ± 5.2	0.413	65.6 ± 6.7	65.5 ± 5.7	0.729	65.5 ± 6.4	66.7 ± 3.5	0.373
E, cm/s	1.0 ± 0.2	1.0 ± 0.2	0.875	1.0 ± 0.2	1.0 ± 0.2	0.911	1.0 ± 0.2	0.9 ± 0.2	0.001
A, cm/s	0.6 ± 0.1	0.7 ± 0.1	0.040	0.6 ± 0.1	0.7 ± 0.1	0.271	0.6 ± 0.1	0.8 ± 0.2	0.001
E/A	1.5 ± 0.4	1.5 ± 0.4	0.147	1.5 ± 0.4	1.5 ± 0.4	0.279	1.6 ± 0.4	1.2 ± 0.3	< 0.001
Em (Lateral), cm/s	17.8 ± 3	16 ± 3.4	< 0.001	17.6 ± 3.1	17.1 ± 3.3	0.097	17.5 ± 3.1	15.4 ± 4.0	0.001
E/Em	5.5 ± 1.2	6.3 ± 1.6	< 0.001	5.6 ± 1.2	5.8 ± 1.4	0.121	5.7 ± 1.3	5.9 ± 1.9	0.376
DT, ms	239.6 ± 66.9	237.4 ± 59.1	0.790	238.7 ± 65.9	238.3 ± 61.9	0.900	237.4 ± 52.1	238.6 ± 64.4	0.852

Values are given as mean ± standard deviation, median (interquartile range) or count (percentage).

*LAD* left atrial diameter, *LAA* left atrial area, *LAVI* left atrium volume index, *RAA* right atrial area, *LVEDA* left ventricular end-diastolic area, *LVEDD* left ventricular end-diastolic dimension, *RV-ap* right ventricular anteroposterior diameter, *RVEDA* right ventricular end-diastolic area, *LVEF* left ventricular ejection fraction, *E* mitral inflow early diastolic velocity, *A* mitral inflow late filling wave velocity, *Em* peak early diastolic myocardial velocity, *DT* deceleration time.

### Echocardiographic indices and maternal age

Pearson correlation analysis showed that maternal age was positively correlated with LAD (*p* < 0.001), LAA (*p* < 0.001), RAA (*p* = 0.004), E/Em (*p* = 0.003) and negatively correlated with E/A (*p* = 0.007), Em (*p* < 0.001). Multiple linear regression analysis was used to further examine the independent effect of maternal age on the echocardiographic parameter (Table 3). We could see maternal age was an independent impact factor of LAD, LAA, Em and E/Em adjusted by maternal age, parity, BMI and heart rate.

**Table 3:** Independent effect of maternal age on the echocardiographic indices.

	Standardized coefficients	R^2^	*p* value
LAD	0.135	0.135	0.045
LAA	0.208	0.183	0.008
Em	− 0.231	0.158	0.001
E/Em	0.153	0.045	0.036

*LAD* left atrial diameter, *LAA* left atrial area, *Em* peak early diastolic myocardial velocity, *E/Em* ratio of Peak velocity blood flow in early diastole to peak early diastolic myocardial velocity.

### Maternal age and cardiac diastolic function

#### Diastolic function between maternal women grouped by age

When diastolic function decline was defined as Em < 13 cm/s (deciles of Em value), it was seen in 7.8% of younger women compared with 28.6% of those in advanced maternal aged group (*p* < 0.001).

By using multiple logistic regression analysis, maternal age and weight were the independent effect factors of diastolic function decline when adjusted by maternal age, parity, BMI and heart rate (Table 4).

**Table 4:** Independent effect of parameters on the prediction of diastolic function decline (Em < 13).

	Hazard ratio (95% CI)	*p*
Age, year	1.133 (1.020–1.257)	0.019
Parity	1.029 (0.383–2.767)	0.954
Gestational age, weeks	0.969 (0.692–1.357)	0.853
BMI	1.125 (1.022–1.240)	0.017
Heart rate	1.026 (0.992–1.061)	0.142

*95% CI* 95% confidence interval, *BMI* body mass index.

#### When did maternal women tend to show diastolic function decline?

**Figure 1:**
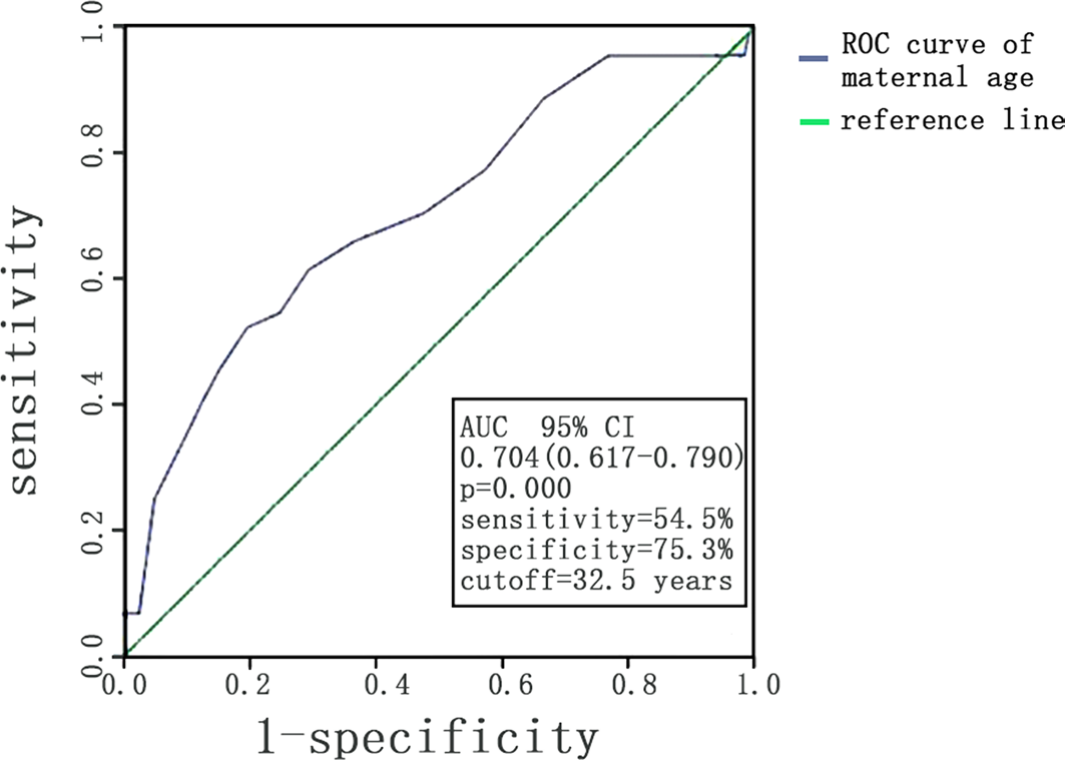
ROC curve analysis of maternal age to differentiate maternal women with cardiac diastolic function decline from those without cardiac diastolic function decline.

By ROC curve analysis, the optimal cut-off value of maternal age to detect women with cardiac diastolic function decline was 32.5 years with an area under the curve value of 0.704 (95% CI 0.617–0.790, p < 0.001), a sensitivity of 54.5% and specificity of 75.3% (Fig. 1).

### Parity and cardiac diastolic function

#### Difference of cardiac diastolic function between nulliparous and primiparous women

**Figure 2:**
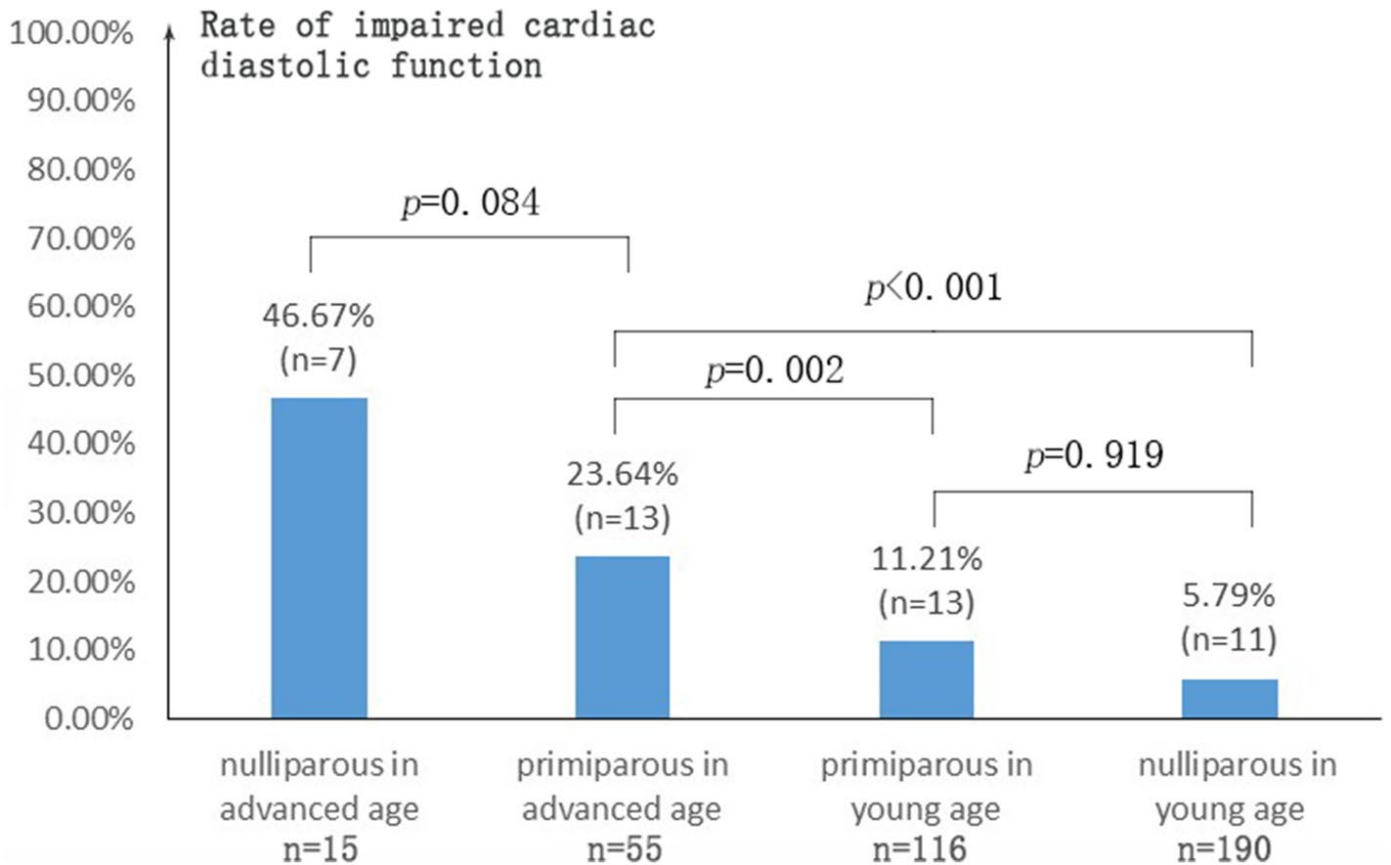
Rate of impaired cardiac diastolic function in different group divided by age and parity. Advanced age was defined as maternal age ≥ 35 years and young age was defined as maternal age < 35 years. Rate of impaired cardiac diastolic function was defined as number of impaired cardiac diastolic function in each group divided by total number of the corresponding group. Kruskal–Wallis test was used to analysis the statistical difference.

There was no difference in the rate of impaired cardiac diastolic function in nulliparous and primiparous women (*p* = 0.054). Kruskal–Wallis test was used to compare the rate of impaired cardiac diastolic function in different groups divided by age and parity (Fig. 2). Independent from parity, the rate of impaired cardiac diastolic function was significantly higher in women of advanced age than that in women of young age. While in both subgroups of advanced age and young age, parity had no significant effect on the rate of impaired cardiac diastolic function.

### Pre-eclampsia occurrence based on Em levels

**Figure 3:**
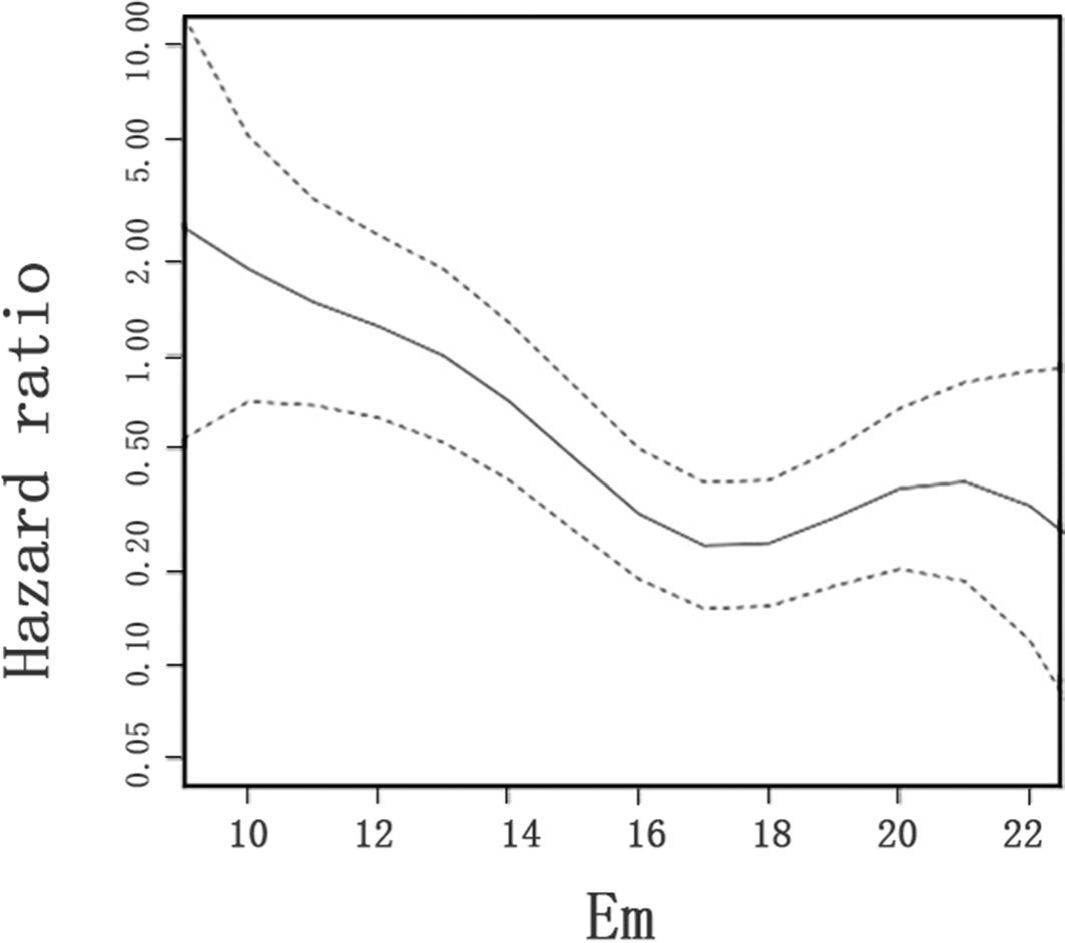
Correlation between Em and the risk of pre-eclampsia among pregnant women. Penalized smoothing spline (P-splines) and univariate cox proportional hazards method were used to assess the tendency of the occurrence rate of pre-eclampsia. Em < 13 cm/s was strongly correlated with pre-eclampsia occurrence (HR 8.56; 95% CI 3.40–21.57) after being adjusted for the confounders (age, parity, gestational week, LVEF, LAVI). Correlation between Em and the risk of pre-eclampsia occurrence was shown in Figure. There was a tendency that larger Em value was associated with lower hazard ratio.

During a median follow-up of 182 (175, 182) days, there were 25 pre-eclampsia occurrence (6.7%) including 14 mild pre-eclampsia and 11 (44%) severe pre-eclampsia. Gestation weeks of onset of pre-eclampsia was 31 (27, 34). Among the pre-eclampsia group, 22 (88%) had proteinuria, 4 (16%) had impaired liver function, 2 (8%) had elevated creatinine and 2 (8%) had reduced platelet. The baseline clinical characteristics and echocardiographic parameter of the pre-eclampsia group was showed in Tables 1 and 2. The pre-eclampsia occurrence was significantly higher in patients with Em < 13 cm/s compared with those with Em ≥ 13 cm/s, and was diagnosed in 22.7% women with impaired diastolic function at mid-gestation while in only 4.5% of those in normal group (*p* < 0.001). Having impaired diastolic function or not had no effect on the severity of preeclampsia (*p* = 0.414). The multivariable COX model was shown in Table 5. Em < 13 cm/s was strongly associated with pre-eclampsia occurrence (HR 8.56; 95% CI 3.40–21.57) after being adjusted for the confounders (Table 5). Correlation between Em and the risk of pre-eclampsia occurrence was shown in Fig. 3. There was a tendency that larger Em value was associated with lower hazard ratio.

**Table 5:** Cox proportional hazard model-multivariable analysis.

	Hazard ratio (95% CI)	*p*
Em < 13 cm/s	8.56 (3.40–21.57)	< 0.001
Age, year	1.02 (0.91–1.15)	0.72
Parity	0.77 (0.27–2.20)	0.63
Gestational age, weeks	0.74 (0.45–1.12)	0.17
LVEF, %	1.03 (0.96–1.12)	0.37
LAVI	0.90 (0.44–1.81)	0.77

*95% CI* 95% confidence interval, *LVEF* left ventricular ejection fraction, *LAVI* left atrium volume index.

## Discussion

### Echocardiographic indices and advanced maternal age

Previous study showed that advanced maternal age is associated with decreased cardiac systolic function as well as cardiac output^8^. This study showed us that cardiac diastolic function, such as LAA, mitral inflow and tissue Doppler (TD) indices, changed depending on maternal age. It should be mentioned that cardiac diastolic function decreases as age increases in non-pregnancy, but it is mostly observed in those who were older than 50 years^11^. In our study, there was a significant higher rate of diastolic function decline in maternal women who were older than 35 years old (28.6% vs. 7.8%) with normal left ventricular ejection fraction, which suggested the susceptibility of diastolic function to cardiac maladaptation of pregnancy in advanced age. ROC curve showed that pregnant women over 32 years old tended to have diastolic function decline, earlier than the traditional advanced age of 35.

Normal diastolic function is actually the ability of the ventricle to fill to a normal end-diastolic volume without an abnormal increase in left ventricular end-diastolic or mean left atrial pressure. Diastolic function is a complex phenomenon that should be assessed by the simultaneous evaluation of cardiac geometric indices and Doppler indices as well as loading conditions^7^. Previous study^7,12^ found that the left atrial enlarged gradually from as early as 5 weeks of gestation and were concomitant with a same tendency in stroke volume (SV) and cardiac output (CO), which was probably due to an increase in preload. Remodeling or enlargement of left and right atrium reflected the cardiac adaptation to increasing preload of maternal women. In this study we found LAA and RAA were higher in advanced age pregnancy, which might indicate a worse tolerance to increasing preload in those women.

As for TD indices, Em wave has been considered to be a measurement of left ventricular diastolic function which is relatively independent of left ventricular filling pressure and a reflection of ventricular active diastole in some degree^13^. We could see maternal age was an independent factor of Em, indicating that the ability of ventricular active diastole is impaired when age increases. When using Em < 13 cm/s (10% of our population) as a criterion of diastolic function decline, we found a significantly larger proportion of that in advanced maternal age women. The underlying pathophysiology that maternal women in advanced age tend to show diastolic dysfunction remains unclear and it may associate with the change of hormone level. Estrogen level is reduced in women of advanced age^14^. Estrogen level of maternal women were thought to closely associated with variance of cardiac structure and function for its influence on the metabolism and remodeling of myocardium by influencing E2-mediated mitochondrial metabolism, enhancing left ventricular hypertrophy and altering cellular calcium homeostasis^15,16^. Besides, recent study showed low plasma estrogens-2 (E2) levels in pre-eclamptic women and suggested estrogen dysregulation could play a key role in pre-eclampsia, which might be associated with cardiac function maladaptation in pre-eclampsia pregnancy^17^.

To sum up, women in advanced age and even those who were over 32 years old were more likely to show cardiac diastolic dysfunction and therefore be exposed to a higher risk of adverse pregnancy outcomes, which remind us of paying more attention to the monitoring of cardiac function and the screening of pregnancy complications in those women.

### Difference of cardiac function adapted to pregnancy between nulliparous and primiparous women

As could be seen in previous study^18^, after adjustment by age, primiparous women had a lower risk of pregnancy complication such as hypertensive disorder, gestational diabetes mellitus or preterm delivery and adverse pregnancy outcome such as stillbirth. However, difference of cardiac function adapted to pregnancy between nulliparous and primiparous women still remains unknown.

Some cardiac dimensions and volumes indices such as LAA, RAA, RVEDA were significantly higher in primiparous women although no significant difference in the rate of impaired diastolic function was found between the two groups. This reflected normal cardiac adaption to pregnancy hemodynamics change. Previous study^19^ showed that the rates of left ventricular diastolic dysfunction were not so different between nulliparous women and multiparous women (parity from 0 to 4). It was also demonstrated in some studies that among advanced age maternal women, the rate of hypertensive disorder and fetal growth restriction was higher in nulliparous women^20^, the incidence of perinatal complication such as preterm delivery and low birth weight infant was more likely to increase with age in nulliparous women^21^, which is consistent with the hypothesis that primiparous women can better tolerate advanced-age pregnancy. However, the pathophysiology of this difference have not yet been identified. Previous study showed that nulliparous women had a significantly higher level of circulating soluble fms-like tyrosine kinase (sFlt-1) in their second pregnancy, which is an inhibitor of placental growth factor (PLGF) and vascular endothelial growth factor and might contribute to high risk of pre-eclampsia^22^.

### Impaired cardiac diastolic function at mid-gestation is associated with pre-eclampsia occurrence

Hypertensive disorders of pregnancy are the most prevalent complications within gestation, in which pre-eclampsia is relatively severe and closely related with pregnancy outcome^9^. Current studies show that maternal cardiac diastolic dysfunction is associated with pre-eclampsia. Study found that women with term pre-eclampsia (37 to 42 gestation weeks) demonstrated a significantly higher rate of global diastolic dysfunction^23^. Muthyala found that the diastolic dysfunction was associated with the severity of pre-eclampsia, around 40% of women with severe preeclampsia had diastolic dysfunction while only 3.3% of those with mild preeclampsia had diastolic dysfunction^6^. Muthyala’s study showed a lower rate of diastolic dysfunction among pre-eclampsia pregnancy than our study. Since Muthyala’s study only included women below the age of 35, the different result might be caused by the different age of the subjects in two studies. Melchiorre found that 33% women who went on to develop pre-eclampsia earlier than 38 gestation weeks began to demonstrate left ventricular diastolic dysfunction at mid-gestation (20–23 weeks) while 3.1% in normal pregnancy^5^. Our study showed that exhibiting impaired diastolic function at mid-gestation (mostly at 14 weeks) were also associated with high morbidity of pre-eclampsia. It seems that diastolic function change may occur before and alongside the onset of preeclampsia. Women affected by preeclampsia present with high total vascular resistance, sympathetic vasoconstrictor activity, reflecting a significant burden on the heart, which leads to LV remodeling and subsequent systolic or diastolic dysfunction^24^. The observation that Pre-eclampsia was diagnosed in 22.7% women with diastolic function decline at mid- gestation while in only 4.5% of women with normal diastolic function suggested that impaired cardiac diastolic function might be an early indication of cardiovascular disease susceptibility and a significant predictive factor for pre-eclampsia. The mechanism that pregnancy with impaired cardiac function is associated with high morbidity of pre-eclampsia remains unknown. It is believed that pathogenetic mechanisms involved in pre-eclampsia include placental ischemia, defective deep placentation, oxidative and endoplasmic reticulum stress, intravascular inflammation and endothelial dysfunction^25^. Pre-eclampsia appears after almost 20 weeks, while those involved pathogenetic mechanisms may last starting from an early stage, thus leading to a hemodynamics change in maternal circulation, and cardiac diastolic function is sensitive to the change. Toxic substrates from a chronically ischemic placenta and elevated maternal cathecolamines might lead to widespread elevated systemic vascular resistance, endothelial cell damage and increased left ventricular afterload, all of which combined result in left ventricular hypertrophy with impaired ventricular filling manifested as significant diastolic dysfunction^26^.

Interestingly, there were 32 women developing gestational diabetes during the follow-up. 3 (9.4%) women with gestational diabetes developed preeclampsia and 29 (6.4%) women without gestational diabetes developed preeclampsia (p = 0.544). There was no difference of rate of gestational diabetes between Impaired cardiac diastolic function group and normal cardiac diastolic function group (9.1% vs. 8.4%, *p* = 0.833). Previous study^27^ showed that abnormal glucose tolerance was associated with higher A wave but had no influence on E/A ratio or Em, indicating Glucose metabolic disorders might influence cardiac diastolic function. Further study is worth to explore the relation between Glucose metabolism, hypertensive disorder and cardiac function.

## Limitations

Our study was a retrospective, single-center study. This could lead to selection bias. Pregnancy with co-morbidities and non-singleton pregnancies were excluded in this study and further study on their potential impact on diastolic function and occurrence of pre-eclampsia is of worth. The study on the pathophysiology of correlation between cardiac diastolic function decline at mid-gestation and onset of pre-eclampsia is also of worth. Additional measurements of diastolic dysfunction such as left ventricular isovolumetric relaxation time (IVRT) and Global longitudinal strain/ speckle tracing might also be of worth.

## Conclusion

Maternal age was an independent effect factor of diastolic function decline, and maternal women tended to show diastolic dysfunction decline from as early as 32.5-year-old. There was no difference in cardiac diastolic function between nulliparous and primiparous women. Pregnancy with cardiac diastolic dysfunction at gestation week of 14–20 had a significantly higher occurrence rate of pre-eclampsia during the whole gestation. We should pay more attention to advanced-age maternal women and diastolic function decline at mid-gestation. The application of risk grading using diastolic function at mid-gestation may improve the survival outcomes of pregnant women.
